# Apoptotic transition of senescent cells accompanied with mitochondrial hyper-function

**DOI:** 10.18632/oncotarget.8536

**Published:** 2016-04-01

**Authors:** Danli Wang, Yang Liu, Rui Zhang, Fen Zhang, Weihao Sui, Li Chen, Ran Zheng, Xiaowen Chen, Feiqiu Wen, Hong-Wei Ouyang, Junfeng Ji

**Affiliations:** ^1^ Center of Stem Cell and Regenerative Medicine, School of Medicine, Zhejiang University, Hangzhou, China; ^2^ Zhejiang Provincial Key Laboratory of Tissue Engineering and Regenerative Medicine, Hangzhou, China; ^3^ Division of Hematology and Oncology, Shenzhen Children's Hospital, Shenzhen, China

**Keywords:** senescence, metabolism, mitochondria, ROS, apoptosis

## Abstract

Defined as stable cell-cycle arrest, cellular senescence plays an important role in diverse biological processes including tumorigenesis, organismal aging, and embryonic development. Although increasing evidence has documented the metabolic changes in senescent cells, mitochondrial function and its potential contribution to the fate of senescent cells remain largely unknown. Here, using two *in vitro* models of cellular senescence induced by doxorubicin treatment and prolonged passaging of neonatal human foreskin fibroblasts, we report that senescent cells exhibited high ROS level and augmented glucose metabolic rate concomitant with both morphological and quantitative changes of mitochondria. Furthermore, mitochondrial membrane potential depolarized at late stage of senescent cells which eventually led to apoptosis. Our study reveals that mitochondrial hyper-function contributes to the implementation of cellular senescence and we propose a model in which the mitochondrion acts as the key player in promoting fate-determination in senescent cells.

## INTRODUCTION

Cellular senescence, defined as an irreversible arrest of cell proliferation [1], has important functional implications in tumorigenesis and organismal aging [2–3]. Latest studies have also reported that cellular senescence plays non-pathological roles in embryonic development and regeneration [4–7]. Diverse stimuli such as replicative exhaustion, genotoxic stress and oncogene activation are able to trigger cellular senescence [2]. Therefore, depending on the inducers, cellular senescence can be classified as replicative senescence, stress-induced senescence and oncogene-induced senescence (OIS) [2–3]. While replicative senescence is due to telomere attrition, stress-induced and oncogene-induced senescence are independent of telomere shortening therefore being collectively termed as premature senescence. Senescent cells are characterized by a set of biomarkers that include morphological changes, senescence-associated β-galactosidase (SA-β-gal) activity, tumor suppression network activation involving p53 and p16/Rb pathways, senescence-associated heterochromatin foci (SAHF), and senescence-associated secretory phenotype (SASP) [1, 4].

SASP including a diverse array of cytokines, chemokines and proteases endows senescent cells with the ability to exert non-autonomous biological functions through paracrine effects [3]. This robust protein secretion function suggests that the metabolism senescent cells is highly active. Mounting studies have documented that senescent cells induced by various stimuli exhibited profound alterations in their metabolism and bioenergetics. For example, senescent lymphoma cells induced by doxorubicin treatment display a highly glucose-consuming and energy-producing profile [8]. Metabolic and proteomic analysis has shown that OIS cells manifest altered fatty acid metabolism, an anti-Warburg effect of glycolysis and nucleotide deficiency [9–13]. On the other hand, senescence induced by nucleotide deficiency which triggers replication stress can be bypassed by inactivation ATM through restoration of glucose and glutamine metabolism [14], suggesting that metabolic changes are not only the consequences of senescence, but also may play a causative role.

Mitochondrion is a key organelle for metabolism. Respiratory complexes in the mitochondrion produce important co-factors and metabolites not only used in cellular respiration reactions but also required for other essential cellular functions [15]. Studies have shown that mitochondrial dysfunction not only induces cellular senescence but also drives organismal aging through mechanisms implicating mitochondrial reactive oxygen species (ROS), AMPK and etc [16–19]. In addition, studies have also reported the implication of mitochondrial metabolism enzymes like malic enzymes (ME1/2) [20], malate dehydrogenase (MDH1) [21] and pyruvate dehydrogenase (PDH) [22] in cellular senescence.

Mounting studies have correlated mitochondrial elongation with the establishment of cellular senescence. Inhibition of mitochondrial fission promotes their elongation and the establishment of cellular senescence, and processes that stimulate mitochondrial fission have been shown to reduce senescence-associated phenotypic changes [23–25]. During senescence, the fission 1 (FIS1) protein is down-regulated leading to mitochondrial elongation [27–28]. Knockdown of FIS1 [25] or depletion of the membrane associated ring finger C3HC4 5 (MARCH5), a mitochondrial E3 ubiquitin ligase [29], promotes mitochondrial elongation and induction of senescence. Overexpression of FIS1 in senescent cells is able to reverse both mitochondrial elongation and appearance of senescent phenotypes, suggestive of its involvement in the process [27]. Therefore, sustained mitochondrial elongation may promote senescence-associated phenotypic changes that can be reversed by mitochondrial fission.

Despite increasing evidence showing a role of mitochondria in cellular senescence [30–31], the correlation between the morphological change of mitochondria and the metabolic signature of senescent cells remains unexplored. Senescent fibroblasts have been found susceptible to apoptotic signals [32], and ABT263, a specific inhibitor of the anti-apoptotic proteins BCL-2 and BCL-xL, selectively kills senescent cells in culture in a cell type and species-independent manner by inducing apoptosis [33]. Combination of the synthetic nucleoside analog ganciclovir (GCV) with herpes simplex virus thymidine kinase (GCV-HSVtk) was effectively used to promote either the targeted formation or eradication of senescent cells via mitochondrial DNA (mtDNA) damage and caspase-dependent apoptosis, respectively [34]. Although senescent cells have long been thought to be the alternative cellular state to apoptosis [35–36], the final outcome of senescent cells remains to be examined.

In this study, we explored the bio-energetic profiles of therapy-induced senescence (TIS) using doxorubicin treatment and replicative senescence (RS) by prolonged passaging of human foreskin fibroblasts (HFFs), and characterized the morphological and functional changes of mitochondria during senescence. Our results showed that senescent cells display both morphologically and quantitatively changed mitochondria, accompanied by augmented glucose metabolic rate. Our results are consistent with the proposed role of mitochondrial hyper-function and hypertrophy in driving organismal aging [37–38]. Furthermore, we demonstrated that at the late stage of senescence, likely driven by the aggravated cellular oxidative stress, the mitochondrial membrane potential (MMP) became depolarized, which eventually led to apoptosis.

## RESULTS

### Doxorubicin treatment and prolonged passaging induce senescence in HFFs

**Figure 1:**
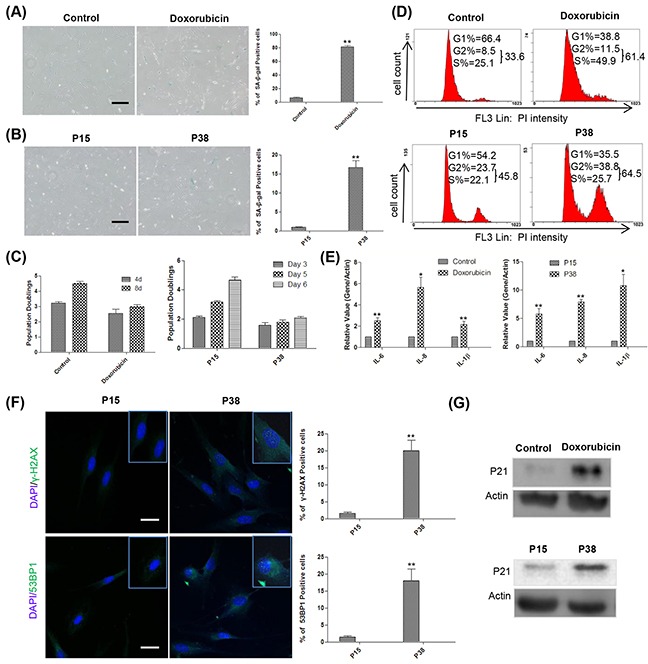
Doxorubicin treatment and replicative passaging induce senescence in HFFs **A.** Left and middle, at day 4, 60 000 control and doxorubicin treated HFFs were reseeded into a 3.5-cm dish for SA-β-gal-staining 12h later. Right, percentages of SA-β-gal-positive cells (**p <0.01, t-test, n = 3). Scale bar, 200 μm. **B.** Left and middle, SA-β-gal-staining of HFFs at P15 and P38. Right, percentages of SA-β-gal-positive cells (**p <0.01, t-test, n = 3). Scale bar, 200 μm. **C.** Left, The numbers of cells treated with or without 100 ng/ml doxorubicin were counted at days 4 and 8, population doublings were calculated and plotted, n = 3. Right, P15 and P38 HFF cells were seeded at 60 000 cells/well into 6-well plates and counted for the indicated times, population doublings were calculated and plotted, n=3. **D.** Up, representative cell cycle analysis of HFFs treated with or without 100 ng/ml doxorubicin at day 4. Down, P15 and P38 HFF cells were seeded at 130 000 cells/6-cm dish and collected 3 days later for flow cytometry, n=3. **E.** Left, gene expression levels (quantitative real-time PCR) in control and doxorubicin-treated HFFs on day 4 (n = 3). Right, gene expression levels in HFFs at P15 and P38 (n = 3). **F.** Left, P15 and P38 cells were stained with by γ-H2AX and 53BP1 antibodies, respectively. Right, the percentages of γ-H2AX and 53BP1 positive cells were quantified (**p < 0.01, t-test, n = 3). Scale bar: 50μm. **G.** Up, representative example of the p21 expression levels in control and doxorubicin-treated HFFs on day 4 (n = 3). Down, p21 levels in HFFs at P15 and P38 (n = 3).

Doxorubicin (Adriamycin), a DNA topoisomerase II inhibitor widely used as a potent antitumor drug [39], has been reported to induce a senescence-like phenotype known as tumor suppression therapy-induced senescence (TIS) [40]. In this study, we found that treatment with doxorubicin at 100 ng/ml for 12 hours induced morphological changes and SA-β-gal activity at day 4 (Figure 1A). Pretreatment with ICRF 187, another DNA topoisomerase II inhibitor [41], partially blocked the effect of doxorubicin (Supplementary Figure S1), suggesting that doxorubicin exerted the effect primarily through inhibiting topoisomerase II. In addition, prolonged passaging of HFFs also led to significantly increased SA-β-gal positivity at as early as passage 38 (P38) (Figure 1B). Cellular proliferation and cell cycle analysis further confirmed that both doxorubicin treatment and replicative passaging resulted in reduced cell proliferation and cell cycle arrest (Figure 1C, 1D). Moreover, the doxorubicin-treated and late-passage cells highly expressed SASP components such as IL-6, IL-8 and IL-1β (Figure 1E). Immunostaining results showed that, in contrast to the control, doxorubicin treated (unpublished data) and P38 HFFs exhibited significantly increased 53BP1 andγ-H2AX foci in the nucleus indicative of DNA damage [42] (Figure 1F). Western blot analysis further demonstrated the up-regulated expression of p21, suggestive of tumor suppression network activation in both doxorubicin-treated and late-passage HFFs [43] (Figure 1G). Taken together, our results showed that doxorubicin treatment and prolonged passaging induce senescence of HFFs.

### Senescent cells exhibited the elevated level of oxidative stress

**Figure 2:**
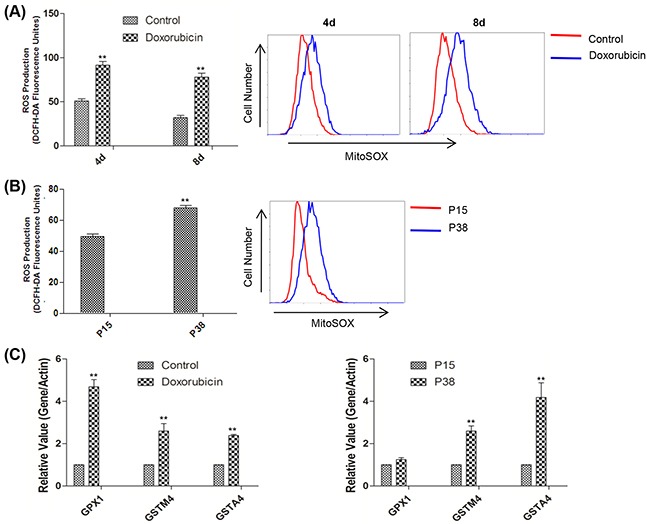
Accelerated oxidative stress was detected in two models of cellular senescence **A.** Left, relative fluorescence intensity of intracellular ROS measured by flow cytometry in control and doxorubicin-treated HFFs on days 4 and 8 (**p <0.01, t-test, n = 3). Right, representative mitochondrial ROS assessment by flow cytometry of MitoSox in control HFFs and HFFs treated with doxorubicin on days 4 and 8. **B.** Left, relative fluorescence intensity of intracellular ROS measured by flow cytometry in HFFs at P15 and P38 (**p <0.01, t-test, n = 3). Right, representative mitochondrial ROS assessed by flow cytometry of MitoSox in HFFs at P15 and P38. **C.** Left, gene expression levels (quantitative real-time PCR) in control and doxorubicin-treated HFFs on day 4 (n = 3). Right, gene expression levels in HFFs at P15 and P38 (**p <0.01, t-test, n = 3).

Since oxidative stress can cause DNA damage which was considered to be a trigger of senescence [44–45], we moved on to evaluate the cellular oxidative state by measuring ROS levels. Compared with the controls, senescent cells induced by both doxorubicin treatment and prolonged passaging possessed higher mitochondrial ROS level as detected by MitoSox [46], which likely contributed to the elevated total cellular ROS levels as measured by a fluorescent probe DCFH-DA (Figure 2A, 2B). Moreover, our qPCR analysis demonstrated the upregulated expressions of antioxidant genes including GPX1 (glutathione peroxidase 1), GSTA4 (glutathione S-transferase A4), and GSTM4 (glutathione S-transferase mu 4) in the doxorubicin-treated and later passage groups relative to the controls, suggesting that oxidative stress induced a defensive anti-oxidative response [47–48] (Figure 2C).

### Senescent cells manifest both morphological and quantitative alterations of mitochondria

**Figure 3:**
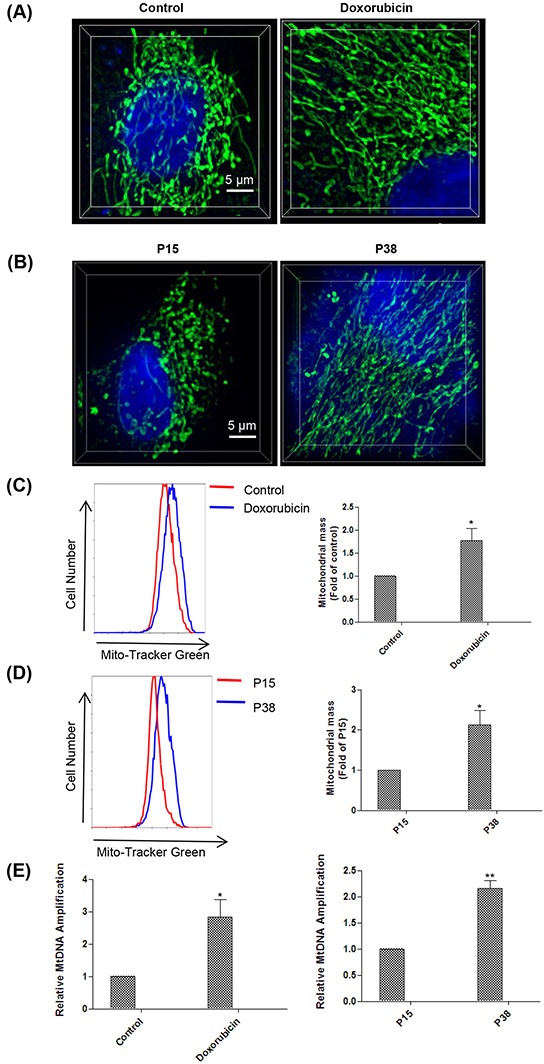
Senescent cells manifest mitochondria alteration both morphologically and quantitatively **A.** Mitochondria morphology observation with structured illumination microscopy (SIM) technology in control and doxorubicin-treated HFFs on day 4 after stained with Mito-Tracker Green and Hoechst 33342 (n = 3). **B.** Mitochondria morphology observation with SIM technology in HFFs at P15 and P38 after stained with Mito-Tracker Green and Hoechst 33342 (n = 3). **C.** Fluorescence intensity assessment by flow cytometry in control and doxorubicin-treated HFFs on day 4 after stained with Mito-Tracker Green (*p < 0.05, t-test, n = 3). **D.** Fluorescence intensity assessment by flow cytometry in HFFs at P15 and P38 after stained with Mito-Tracker Green (*p < 0.05, t-test, n = 3). **E.** Left, increases in mtDNA copy number in doxorubicin-treated HFFs on day 4 compare with controls (*p < 0.05, t-test, n = 3). Right, increases in mtDNA copy number in HFFs at P38 compare with P15 (** *p* < 0.01, t-test, n = 3).

Mitochondrion is the powerhouse of the cell, generating chemical energy in the form of ATP to fuel the activities of the cell. Mitochondrial ROS are produced as a byproduct of ATP generation by oxidative phosphorylation due to electron leakage [49–51]. It prompted us to evaluate the mitochondrial modification in senescent cells induced by doxorubicin treatment and prolonged passaging. To examine the morphological changes of mitochondria in senescent cells, we stained the cells with Mito-Tracker Green, a mitochondrial fluorescent probe. We found that mitochondria in the senescent cells induced by both doxorubicin treatment and prolonged passaging exhibited much more enlarged and elongated morphology compared to that of the controls (Figure 3A, 3B). Moreover, flow cytometry analysis showed that the mitochondrial mass increased in the senescent cells compared to the controls (Figure 3C, 3D), which was confirmed by the increased mitochondrial DNA (mtDNA) copy number as shown by PCR analysis (Figure 3E). Taken together, our results showed that senescent cells exhibited increased number of mitochondria with distinct morphological features.

### Senescent cells harbored hyper-functional mitochondria

**Figure 4:**
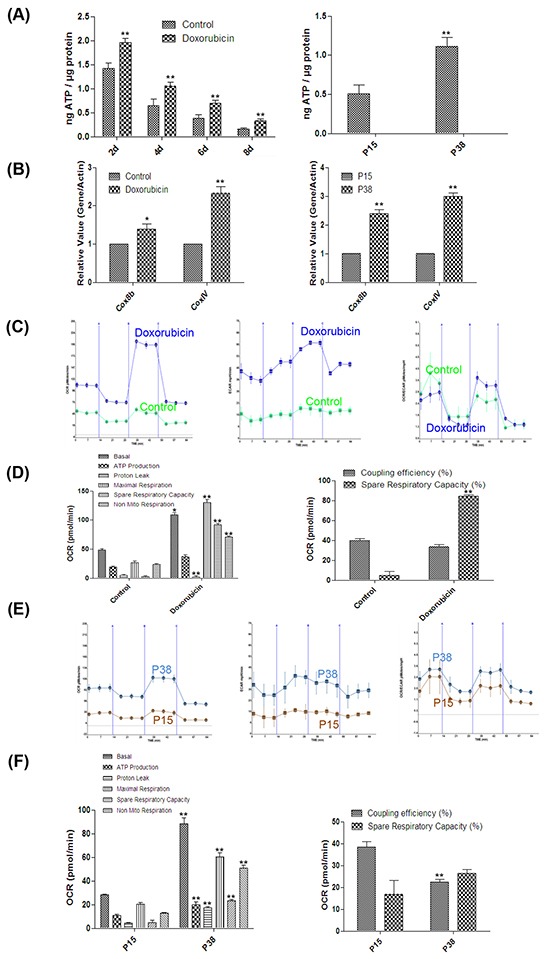
Hyper-functional mitochondria in two models of cellular senescence **A.** Left, ATP concentrations in control and doxorubicin-treated HFFs on days 2, 4, 6 and 8 (**p <0.01, t-test, n = 3). Right, ATP concentrations in HFFs at P15 and P38 (**p <0.01, t-test, n = 3). **B.** Left, gene expression levels (quantitative real-time PCR) in control and doxorubicin-treated HFFs on day 4 (*p < 0.05, **p <0.01, t-test, n = 3). Right, gene expression levels in HFFs at P15 and P38 (**p <0.01, t-test, n = 3). **C.** Cellular respiration in control and doxorubicin-treated HFFs on day 4. Twelve OCR and ECAR measurements were taken over 2 h (3 basal respirations, 3 oligomycin-sensitive respiration, 3 maximal respiratory capacities after FCCP, and 3 non-mitochondrial respirations after antimycin-A and rotenone); the X-axis describes the measurement number. **D.** Basal respiratory capacity, ATP production, proton leak, maximal respiratory capacity, spare respiratory capacity (%), non-mitochondrial respiration and coupling efficiency (%) of doxorubicin-treated HFFs on day 4 compared with control (*p < 0.05, **p <0.01, t-test, n = 3). **E.** Cellular respiration of HFFs at P15 and P38 analyzed as in (C). **F.** Basal respiratory capacity, ATP production, proton leak, maximal respiratory capacity, spare respiratory capacity (%), non-mitochondrial respiration and coupling efficiency (%) of HFFs P38 compared with P15 (**p <0.01, t-test, n = 3).

As the morphology of mitochondria is highly correlated with their functions [52], we went on to explore the mitochondrial functions in the senescent cells in these two models. We first measured the concentrations of ATP, one of the major functions of mitochondria. The ATP concentration in the doxorubicin-treated cells was significantly higher than that of the untreated cells at as early as day 2 and the increase sustained on day 8 (Figure 4A). The ATP concentration of the cells at P38 was also significantly higher than that of the cells at P15 (Figure 4A). Moreover, consistent with the increased ATP generation, our qPCR analysis demonstrated that the expression levels of key genes involved in mitochondrial oxidative phosphorylation such as COX8b, COXIV were upregulated in senescent cells [53] (Figure 4B).

To further examine the mitochondrial function, we used a Seahorse XF Analyzer to evaluate the metabolic profiles of mitochondrial respiration and glycolysis in both TIS and RS HFFs by assessing the time course of respiration of pyruvate as measured by OCR and ECAR [54–57]. Our results showed that basal respiration rate in doxorubicin-treated cells was significantly higher than that in the controls on day 4 after treatment (Figure 4C, 4D). After addition of oligomycin, an ATP synthase inhibitor [58], doxorubicin-treated cells displayed marked ATP turn-over accompanied by a significantly elevated ECAR level (Figure 4C, 4D). Furthermore, a striking increase in maximal respiratory capacity after addition of the proton ionophore FCCP [59] was observed, which resulted in a substantially higher reserve capacity (OCR_MAXIMAL RESPIRATORY CAPACITY_ - OCR_BASAL RESPIRATION_) in doxorubicin-treated cells (Figure 4C,D). Finally, after addition of the electron transport chain inhibitors antimycin-A and rotenone [60–61], doxorubicin-treated cells exhibited a remarkable decrease in mitochondrial respiration compared with the controls (Figure 4C, 4D). Similar results were also found in RS cells at P38 compared with control cells at P15 (Figure 4E, 4F), except that senescent cells in RS models were more prone to TCA cycle, whereas doxorubicin-treated cells showed stronger aerobic glycolysis dependency at basal respiration phase as indicated by OCR/ECAR (Figure 4C, 4E). Consistent with the ATP concentration results, the respiration rate of doxorubicin-treated cells on day 8 after treatment was also higher than that of the control (Supplementary Figure S2). Altogether, our results demonstrated that possibly driven by the increased number, mitochondria in senescent cells exhibited hyper-function, which could exacerbate the oxidative stress.

### Late-stage senescent cells converted to undergoing mitochondria-associated apoptosis

**Figure 5:**
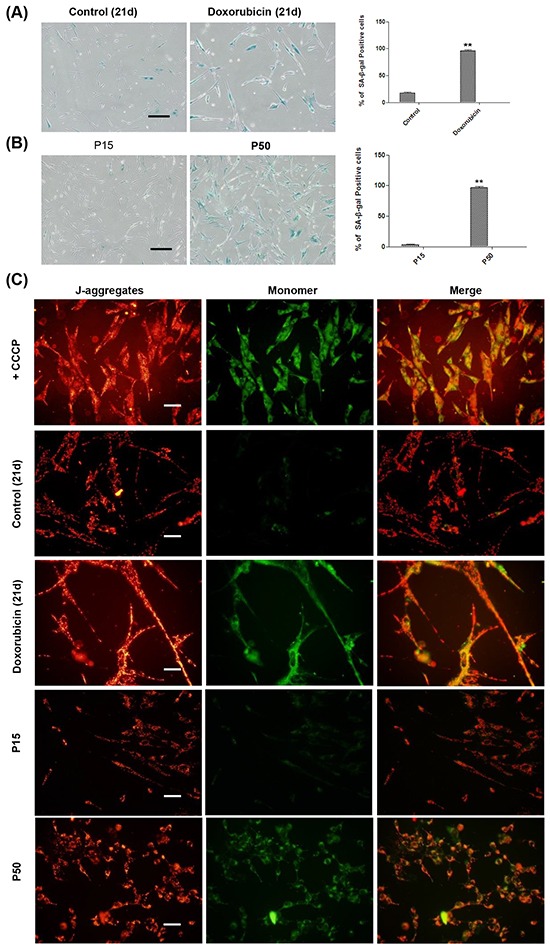
MMP depolarization detection at the late stage of senescence in TIS and RS cells **A.** Left and middle, control and doxorubicin treated HFFs were seeded at day 21 for SA-β-gal-staining. Right, percentages of SA-β-gal-positive cells (**p <0.01, t-test, n = 3). Scale bar, 200 μm. **B.** Left and middle, SA-β-gal-staining of HFFs at P15 and P50. Right, percentages of SA-β-gal-positive cells (**p <0.01, t-test, n = 3). Scale bar, 200 μm. **C.** Representative examples of the fluorescence pattern after staining with JC-1 in TIS cells on day 21 and RS HFFs at P15 and P50. CCCP, an apoptosis inducer used as a positive control. Left, red fluorescence emission under green excitation corresponding to J-aggregates. Middle, green fluorescence emission under blue excitation which corresponding to J-monomers. Right, JC-1 images were merged by Image-J. Scale bar, 50 μm. n = 3.

As the mitochondrion is prone to oxidative damage [62–63], we suspected that as senescence advances, deteriorated oxidative stress may damage the mitochondria, which could lead to apoptosis. We first examined the senescence status of the cells in both TIS and RS models at the very late stage. We found that in TIS, more than 95% of doxorubicin-treated cells displayed SA-β-gal staining positivity on day 21 after treatment (Figure 5A), and almost 100% of the cells at P50 were SA-β-gal staining positive (Figure 5B). It suggests that cells in the cultures of the two models advanced to a full senescent status at this stage.

The mitochondrial membrane potential (MMP), a consequence of the electrochemical proton gradient maintained for the purpose of ATP synthesis [64–65], is also an important indicator of mitochondrial function. Using the membrane-permeable dye JC-1, which indicates a high MMP by the formation of red fluorescent J-aggregates, at the same time displays MMP depolarization by forming a green fluorescent J-monomer [66], we further examined mitochondrial function during the progression of cellular senescence in these two models. Although on day 14, no obvious MMP depolarization was observed (Supplementary Figure S3), doxorubicin-treated cells on day 21 displayed strong green fluorescence of mitochondrial membrane in contrast to the red fluorescent signals in the untreated control (Figure 5C). In TIS model, while MMP depolarization was not observed at P16 or P45 (Supplementary Figure S3), cells at P50 exhibited MMP depolarization as opposed to the cells at P15 (Figure 5C). Taken together, our results demonstrated depolarized MMP in the mitochondria at the late stage of cellular senescence.

**Figure 6:**
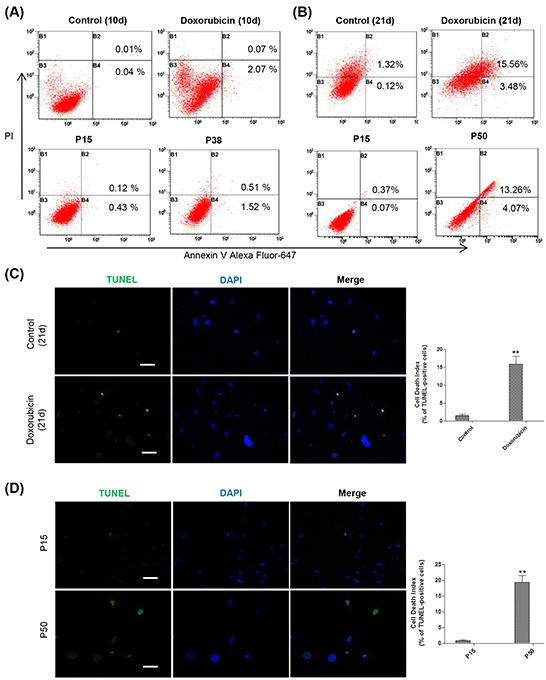
Mitochondria-associated apoptosis occurs at the late stage of cellular senescence **A.** Up, representative apoptosis assessment by flow cytometry in control and doxorubicin-treated HFFs on day 10, stained with Annexin V and PI (n = 3). Down, representative apoptosis assessment in RS HFFs at P15 and P38 analyzed as before (n = 3). **B.** Up, representative apoptosis assessment by flow cytometry in control and doxorubicin-treated HFFs on day 21, stained with Annexin V and PI. Down, apoptosis assessment in RS HFFs at P15 and P50 analyzed as before (n = 3). **C.** DNA fragmentation detected by TUNEL in control and doxorubicin-treated HFFs on day 21. Cells were stained with FITC-12-dUTP labeling mix and recombinant TdT enzyme along with DAPI. Apoptotic cells were visualized as green, and the nuclei as blue (**p <0.01, t-test, n = 3). Scale bar, 100 μm. **D.** DNA fragmentation detected by TUNEL in HFFs at P15 and P50 analyzed as in (C) (**p <0.01, t-test, n = 3). Scale bar, 100 μm.

As MMP has been reported to regulate matrix configuration and cytochrome c release during apoptosis [67–68], we further looked into apoptosis at late stage of cellular senescence. Flow cytometry analysis showed that a considerable proportion of apoptosis was detected in both senescent models at the late stage versus the early stage (Figure 6A, 6B). TUNEL assay further confirmed a high apoptosis rate in the late-stage senescent cells as well (Figure 6C, 6D). Altogether, our results suggested that the depolarized MMP in the mitochondria converted late-stage senescent cells to undergoing apoptosis.

## DISCUSSION

Mounting studies have shown the important role of mitochondria in cellular senescence, and senescent cells show marked metabolic and bio-energetic alterations [8–12, 69–70]. Several studies have demonstrated that sustained mitochondrial elongation may promote senescence-associated phenotypic changes [27–29, 71]. However, how mitochondria associated morphological and metabolic changes happen in senescent cells is not clear. Here, using two in vitro models of cellular senescence induced by doxorubicin treatment and prolonged passaging of neonatal human foreskin fibroblasts, we report that senescent cells exhibited high ROS level concomitant with the mitochondria in senescent cells displays morphological change of giant and elongate and quantitatively alteration as well as elevated glucose metabolic rate. Our results support the mitochondrial hyperfunction theory of aging [37–38].

Mitochondria have long been connected with both apoptosis and cellular senescence. Mitochondrial DNA damage induced apoptosis in senescent cells has been previously reported [34]. Although senescent cells have been thought to be an alternative cellular state to apoptosis, the fate of senescent cells has rarely been tracked. In our study, we found that hyper-functional mitochondria could be both the accomplice and victim of elevated ROS in senescent cells. Together they formed a vicious cycle which led to damaged mitochondria with a depolarized MMP at late stage of senescence and finally induced apoptosis of senescent cells. Our study reveals that mitochondrial hyper-function contributes to the implementation of cellular senescence and we propose a model in which the mitochondrion acts as the key player in promoting fate-determination in senescent cells. It supports the mitochondrial hyper-functional theory of organismal aging [72].

Doxorubicin at a high concentration is known to induce apoptosis [39], but the concentration we used here did not cause evident apoptosis in HFFs even 10 days after treatment (Figure 6A). Our results showed that, despite elevated metabolic rate, senescent cells neither developed impaired MMP nor underwent obvious apoptosis until the very late stage (Figure 5 and Figure 6). It suggests that along with the progression of senescence, the accumulated damage to mitochondria could be responsible for the cell fate transition.

Understanding the energy metabolism of senescent cells will provide insights into understanding the molecular mechanisms regulating cellular senescence, and targeting the metabolism of senescent cells may be able to control both the implementation of cellular senescence and the fate of senescent cells. In this study, we confirmed the involvement of mitochondria in senescence and their contribution to its implementation. Although the molecular mechanisms behind hyper-functional mitochondria have not been explored in this study, our results support the mitochondrial hyperfunction theory of organismal aging suggest that pathways involved in should be targeted for prospective treatment [73]. We also propose a model in which the reciprocal influence of ROS and hyper-functional mitochondria may play a key role in determining the fate of senescent cells. Base on this, strategies aimed at overloading mitochondria might be applicable to accelerate the elimination of senescent cells by inducing their apoptosis.

## EXPERIMENTAL PROCEDURES

### Cell culture and treatment

Neonatal human foreskin fibroblasts (HFFs) from the ATCC were maintained in DMEM high-glucose medium (Corning) supplemented with 10% HyClone fetal bovine serum (Thermo Scientific) at 37°C under 5% CO_2_. In the TIS model, cells were seeded at 1×10^3^/cm^2^ in 10-cm culture dishes. Twenty-four hours after seeding, cells were incubated in complete medium with or without 20 μM ICRF-187 (Selleck Chemicals) for 24h and then the media was changed. Cells were incubated in complete medium with or without 100 ng/ml doxorubicin (Sigma-Aldrich) for 12 h, cultured in fresh complete medium and recorded as day 0. Cells were then prepared for experiments with regular medium changes at different time points. RS was induced by serial passaging of HFFs at a ratio of 1:3 and seeded at ~6 000 cells/cm^2^ in 10-cm dishes every 4-7 days.

### SA-β-gal staining

Cells were counted and seeded onto 6-well plates at 55 000 cells/well before staining. About 12 h after seeding, cells were fixed in 0.2% glutaraldehyde for 10 min at room temperature, washed twice with phosphate-buffered saline (PBS) for 5 min each time, and then stained with X-gal staining solution (1 mg/ml X-gal, 40 mmol/l citric acid/sodium phosphate, 5 mmol/l potassium ferricyanide, 5 mmol/l potassium ferrocyanide, 150 mmol/l NaCl, 2 mmol/l MgCl_2_) at pH 6.0 overnight. Images were captured with an Olympus IX71 microscope (10× magnification). SA-β-gal-positive cells were counted in 3-5 randomly selected images and the percentages of SA-β-gal-positive cells were averaged for statistical analysis.

### Cell cycle analysis

About 2 × 10^6^ cells were fixed with cold 70% ethanol for 1h at 4°C. Cells were then incubated in freshly prepared staining solution consisting of 0.1% Triton X-100 (Sigma), 5 ug/ml PI, and 50 ug/ml DNAse-free RNAse A (Sigma) for 30 minutes at 37°C. Cells were then analyzed on flow cytometer. Cell cycle distribution was determined with the MultiCycle AV-DNA Analysis program.

### Population doubling assay

In TIS model, 55 000 cells were plated in 10-cm dish and treated as before. Then cells were collected at different time points and counted by a hemocytometer. Population doubling levels (PDL) were calculated using the equation PD = log(Nf/N0)/log2 where Nf equals the number of final cells and N0 equals the number of initial cells. Data were expressed as cumulative PDL from 3 independent experiments. In RS models, 330 000 cells were seeded in 10-cm dishes and counted at different time points, then PDL were calculated as in TIS models.

### Immunocytochemistry

Cells were fixed with 4% formaldehyde for 30 minutes, washed with PBS for 5 min twice, and then blocked with PBS containing 1% BSA and 1% triton for 1h at room temperature. Cells were then incubated with polyclonalγ-H2AX (Cell signaling), 53BP1 (a gift from Dr. Jun Huang's laboratory at Life Science Institute, Zhejiang University) antibodies diluted in PBS containing 1% BSA for 2h at room temperature. Cells were then washed with PBS and stained with a DyLight 488 goat anti-rabbit secondary antibody at 1/500 dilution. Nuclei were counterstained with DAPI (5 ug/ml; Beyotime, China). Images were captured with a laser scanning confocal microscope (Olympus DP70).

### Quantitative real-time PCR

cDNA was prepared from total RNA using a Reverse Transcription Kit (Takara). Quantitative real-time PCR was performed using 2×SIBR real-time PCR Pre-mixture (Takara) under the following conditions: 3 min at 95°C followed by 40 cycles at 95°C for 10 s, 55°C for 30 s, and 50°C for 30 s using a CFX-Touch (Bio-Rad) sequence detection system. Data were normalized to the expression of a control gene (β-actin) for each experiment. Data represent the mean ± SD of three independent experiments.

The following primer pairs were used for quantitative real-time PCR:

IL-6: 5ʹ-GGTACATCCTCGACGGCATCT-3ʹ, 5ʹ-GTG CCTCTTTGCTGCTTTCAC-3ʹ;

IL-8: 5ʹ-ATGACTTCCAAGCTGGCCGT-3ʹ, 5ʹ-TCCT TGGCAAAACTGCACCT-3ʹ;

IL-1β: 5ʹ-CCGCCTCAGCCTCCCAAAG-3ʹ, 5ʹ-GC AGTCTACACAGCTTCGGG-3ʹ;

GPX1: 5ʹ-CGGCCCAGTCGGTGTATGC-3ʹ, 5ʹ-CGT GGTGCCTCAGAGGGAC-3ʹ; GSTA4: 5ʹ-AGTTGTACA AGTTGCAGGATGG-3ʹ, 5ʹ-CAATTTCAACCATGGGC ACT-3ʹ;

GSTM4: 5ʹ-TCATCTCCCGCTTTGAGG-3ʹ, 5ʹ-CAGACAGCCACCCTTGTGTA-3ʹ.

### Measurement of intracellular ROS

Cells were incubated at 37°C in the dark with 10μmol/l DCFH-DA diluted with serum-free DMEM for 10-15 min according to the manual from a ROS determination kit (Beyotime, China). Cells were then washed 5 times with PBS, harvested by trypsinization, and collected into 15-ml tubes by centrifugation (5 min at 200×g) at room temperature with aspiration of the supernatant. Then the single-cell suspension was prepared and analyzed by flow cytometry (Cytomics FC 500 MCL, Beckman Coulter) at excitation/emission of 488/525 nm.

### Measurement of mitochondrial ROS

Cells were incubated with 5 μM MitoSox Red, a mitochondrial superoxide indicator (Life Technology, M36008) for 30 min at 37°C in the dark. Cells were then washed three times in warm buffer, and a single-cell suspension was prepared and analyzed by flow cytometry (Cytomics FC 500 MCL, Beckman Coulter) at excitation/emission of 510/580 nm.

### Mitochondrial labeling for morphology observation and mass measurement

Cells were counted and 130 000 cells were then seeded into a 3.5-cm dish 12 h before labeling. For mitochondrial morphology observation, cells were stained by incubation with Hoechst 33342 (Yeasen, China) for 10 min, after washed with three times in PBS, cells were then incubated with 1 μM Mito-Tracker Green (Life Technology, M-7514) for 10 min and observed at 350nm and 490 nm under a structured illumination microscopy (SIM). For mitochondrial mass detection, cells were collected and fluorescence intensity was analyzed by flow cytometry.

### Mitochondrial DNA copy number quantification

DNA was isolated with the QIAamp DNA Mini Kit as described for cell extraction. Mitochondrial DNA content was measured by a multiplex real-time PCR method using an Applied Biosystems 7500 real-time PCR System (Applied Biosystems, Foster City, CA) with the final DNA concentration of 50 ng/μl. Data were normalized to the expression of a control gene (β-actin) for each experiment. Data represent the mean ± SD of three independent experiments. The following primer pairs were used for quantitative real-time PCR:

MT-ND1:

5ʹ- CCCTAAAACCCGCCACATCT-3ʹ, 5ʹ- GAGC GATGGTGAGAGCTAAGGT-3ʹ;

Actin:

5ʹ- AGAGCTACGAGCTGCCTGAC-3ʹ, 5ʹ- AG CACTGTGTTGGCGTACAG-3ʹ.

### ATP concentration assay

Cells in the TIS model were seeded at 1×10^3^/cm^2^ in 10-cm culture dishes. Forty-eight hours after seeding, cells were incubated in complete medium with or without 100 ng/ml doxorubicin (Sigma-Aldrich) for 12 h and then cultured in fresh complete medium with regular medium changes. RS was induced by serial passaging of HFFs at a ratio of 1:3 and seeded at ~6 000 cells/cm^2^ in 10-cm dishes every 4-7 days. For ATP concentration assay, medium of the cells at different time points was removed from the dishes and the cells were washed three times in ice-cold sterile PBS. The plates were incubated at 37°C for 5 min after each wash. Cells were scraped off the surface of the plates in 1 ml of PBS with plastic cell scrapers. The cell suspension was homogenized by repeated pipetting, and the entire volume of each dish was collected. The measurement of ATP concentrations was performed according to the instructions provided with the ATP luminescence kit (FL-ASC, Sigma-Aldrich, St. Louis, MO), and normalized to protein content. Statistical significance was calculated using the two-tailed Student's t-test.

### Metabolic flux analyses

HFFs were cultured on XF-96 plates (Seahorse BioSciences, Billerica, MA, USA) at 6 000 cells/well. On the day of metabolic flux analysis, culture medium was changed to Krebs-Henseleit buffer (25 mmol glucose and 1 mmol pyruvate) at pH 7.4, and incubated at 37°C in a non-CO_2_ incubator for 1 h. All medium and injection reagents used in assays were adjusted to pH 7.4. Using the XF96 metabolic analyzer (Seahorse BioSciences), baseline measurements of oxygen consumption rate (OCR) and extracellular acidification rate (ECAR) were sampled prior to sequential injection of the mitochondrial inhibitors oligomycin (1 μM), FCCP (carbonyl cyanide 4-(trifluoromethoxy)-phenylhydrazone) (0.8 μM), antimycin-A (5 μM), and rotenone (1 μM). OCR and ECAR were automatically calculated and recorded by the Seahorse software. After the assays, the protein level was determined for each well to confirm equal cell density per well.

### Assessment of mitochondrial membrane potential variation

Cells were counted and 60 000 cells were seeded into each well of 6-well plates 12 h before experiments. After three washes with PBS, a JC-1 (5,5ʹ,6,6ʹ-tetrachloro-1,1ʹ,3,3ʹ-tetraethyl-benzimidazolyl carbocyanine iodide) Kit (Beyotime, China) was used to assess the MMP by observation under a fluorescence microscope. For CCCP (Carbonyl cyanide 3-chlorophenylhydrazone) (Yeasen, China) treatment for positive control, the final concentration of 50 μM CCCP was used to incubate cells for 6 h before JC-1 assessment.

### Annexin V for apoptosis assay

Apoptosis was measured by flow cytometry after the cells were harvested and stained with Annexin V conjugated with Alexa Fluor-647 and propidium iodide according to the protocol from the kit (Life Technology, A23204). Data are expressed as the percentages of Annexin V-positive and PI-negative cells (early apoptosis) and as the percentage of both Annexin V-positive and PI-positive cells (late apoptosis or necrosis).

### TUNEL (terminal deoxynucleotidyl transferase (TdT)-mediated dUTP nick-end labeling) assay

Cultured HFFs were counted and 60 000 cells were seeded on cover slips in each well of 6-well plates 12 h before experiments. After three washes with PBS, apoptotic cells *in situ* were identified and quantified at the single-cell level according to the manufacturer's instructions from a cell death detection kit (Roche Diagnostics GmbH, Mannheim, Germany). Then cells were rinsed with PBS, and the nuclei were stained with DAPI (Roche Diagnostics GmbH, Mannheim, Germany) for 5 min at room temperature. The samples were then observed using a fluorescence microscope. In the quantitative analysis, the ratio of apoptotic cells (TUNEL-positive) to total cells (DAPI-stained nuclei) was calculated using four random visual fields from each group. The images were quantified and analyzed using Image J.

### Statistical analyses

The results are presented as the mean ± SEM of a minimum of three independent experiments. Statistical significance was determined by Student's t-test. Differences of *p* <0.05 were considered significant.

## SUPPLEMENTARY FIGURES



## Abbreviations

HFF: human foreskin fibroblast
ROS: reactive oxygen species
MMP: mitochondrial membrane potential
TCA: tricarboxylic acid
ATP: adenosine triphosphate
TIS: therapy-induced senescence
RS: replicative senescence
SIM: structured illumination microscopy
OCR: oxygen consumption rate
ECAR: extracellular acidification rate
FCCP: carbonyl cyanide 4-(trifluoromethoxy)-phenylhydrazone
GPX1: glutathione peroxidase 1
GSTA4: glutathione S-transferase A4
GSTM4: glutathione S-transferase mu 4
