# Supplementary material for: Apoptotic transition of senescent cells accompanied with mitochondrial hyper-function
Source: Oncotarget. 2016 Apr 1;7(19):28286–300. doi: 10.18632/oncotarget.8536 (PMC5053727; doi:10.18632/oncotarget.8536)
Supplement: Supplementary file 1 [file oncotarget-07-28286-s001.pdf]

SUPPLEMENTARY FIGURES

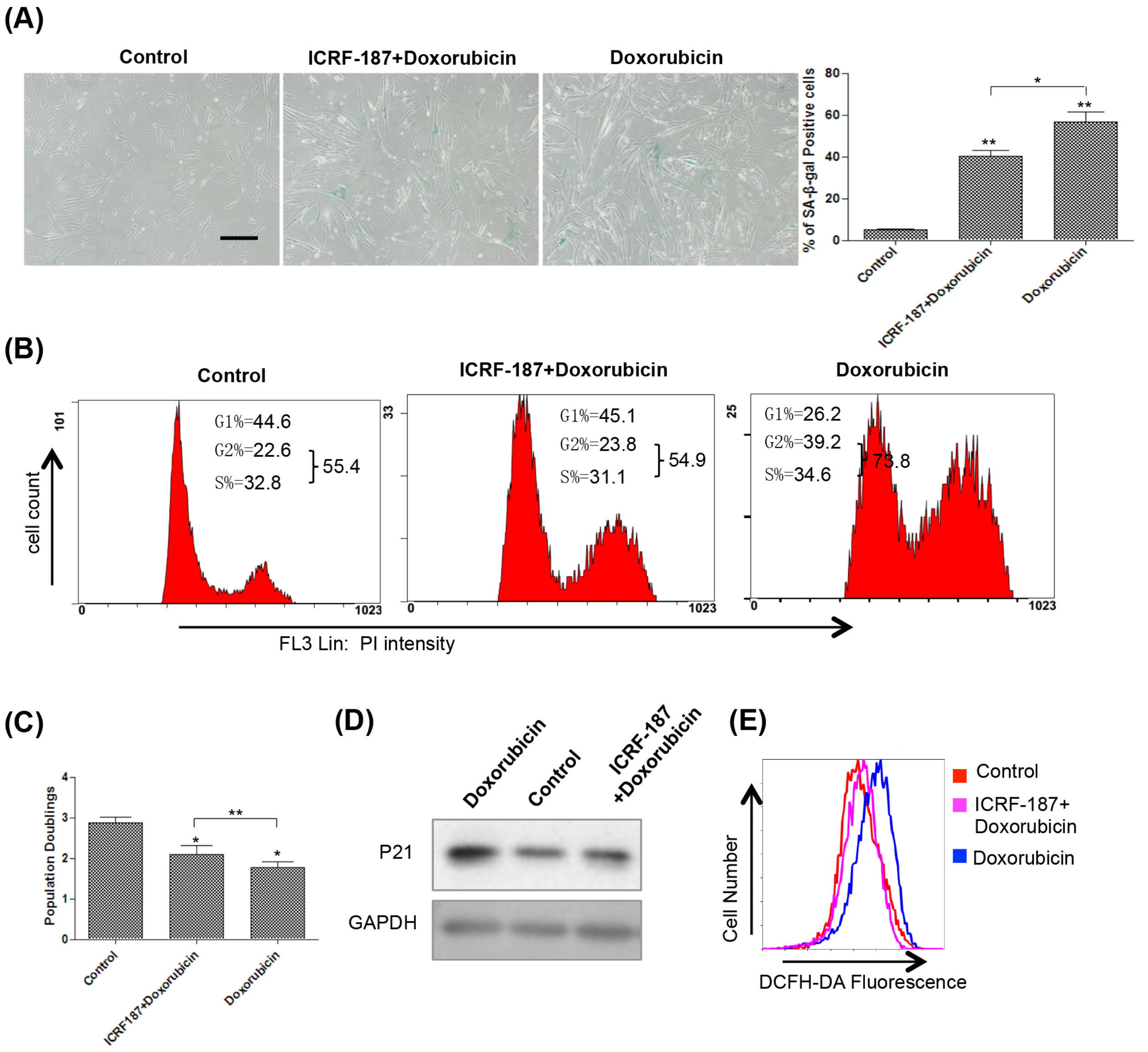

Supplementary Figure S1: ICRF-187 pretreatment partially rescued senescent phenotype induced by doxorubicin. A. Left, control and TIS cells treated with or without ICRF-187 were reseeded at day 3 for SA-β-gal-staining. Right, percentages of SA-β-gal-positive cells (***p* <0.01, t-test, n = 3). Scale bar, 200 μm. **B.** Cell cycle analysis of control and TIS cells treated with or without ICRF-187 at day 3 (n = 3). **C.** The numbers of control and TIS cells treated with or without ICRF-187 were counted at day 3, population doublings were calculated and plotted (***p* <0.01, **p* <0.05, t-test, n = 3). **D.** p21 expression levels in control and TIS cells treated with or without ICRF-187 on day 3 (n = 3). **E.** Representative flow cytometry of cellular ROS assessment in control and TIS cells treated with or without ICRF-187 on day 3 (n = 3).

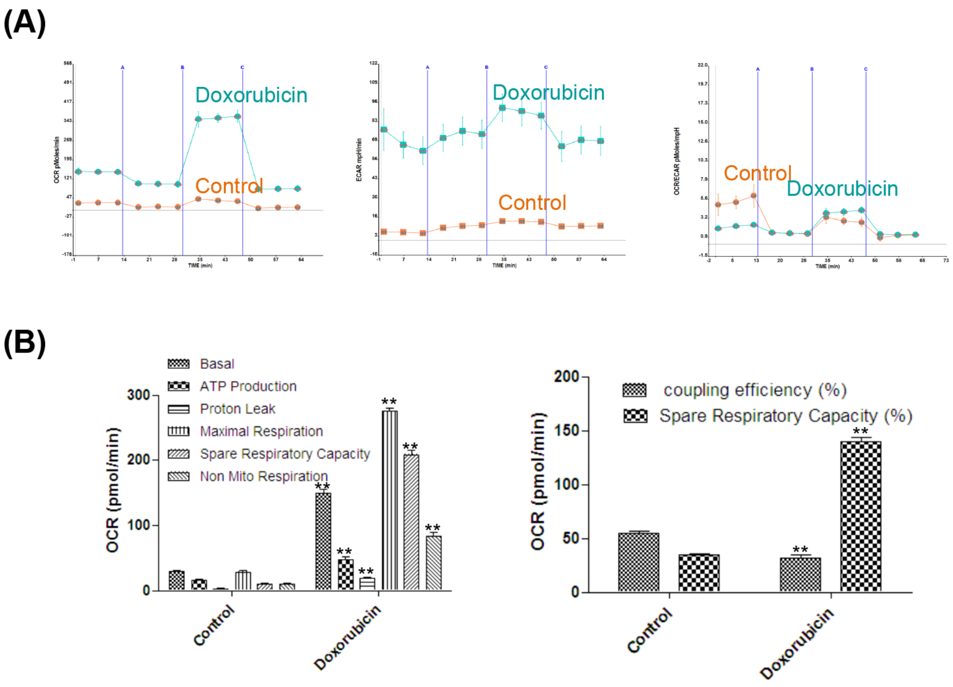

Supplementary Figure S2: Metabolic phenotypes of control and doxorubicin-treated HFFs on day 8. A. Twelve OCR and ECAR measurements were taken over 2 h (3 basal respiration, 3 oligomycin-sensitive respiration, 3 maximal respiratory capacity after FCCP, and 3 non-mitochondrial respiration after antimycin-A and rotenone); the X-axis describes the measurement number. **B.** Basal respiratory capacity, ATP production, proton leak, maximal respiratory capacity, spare respiratory capacity (%), non-mitochondrial respiration and coupling efficiency (%) of doxorubicin-treated HFFs on day 8 compared with control (***p* <0.01, t-test, n = 3).

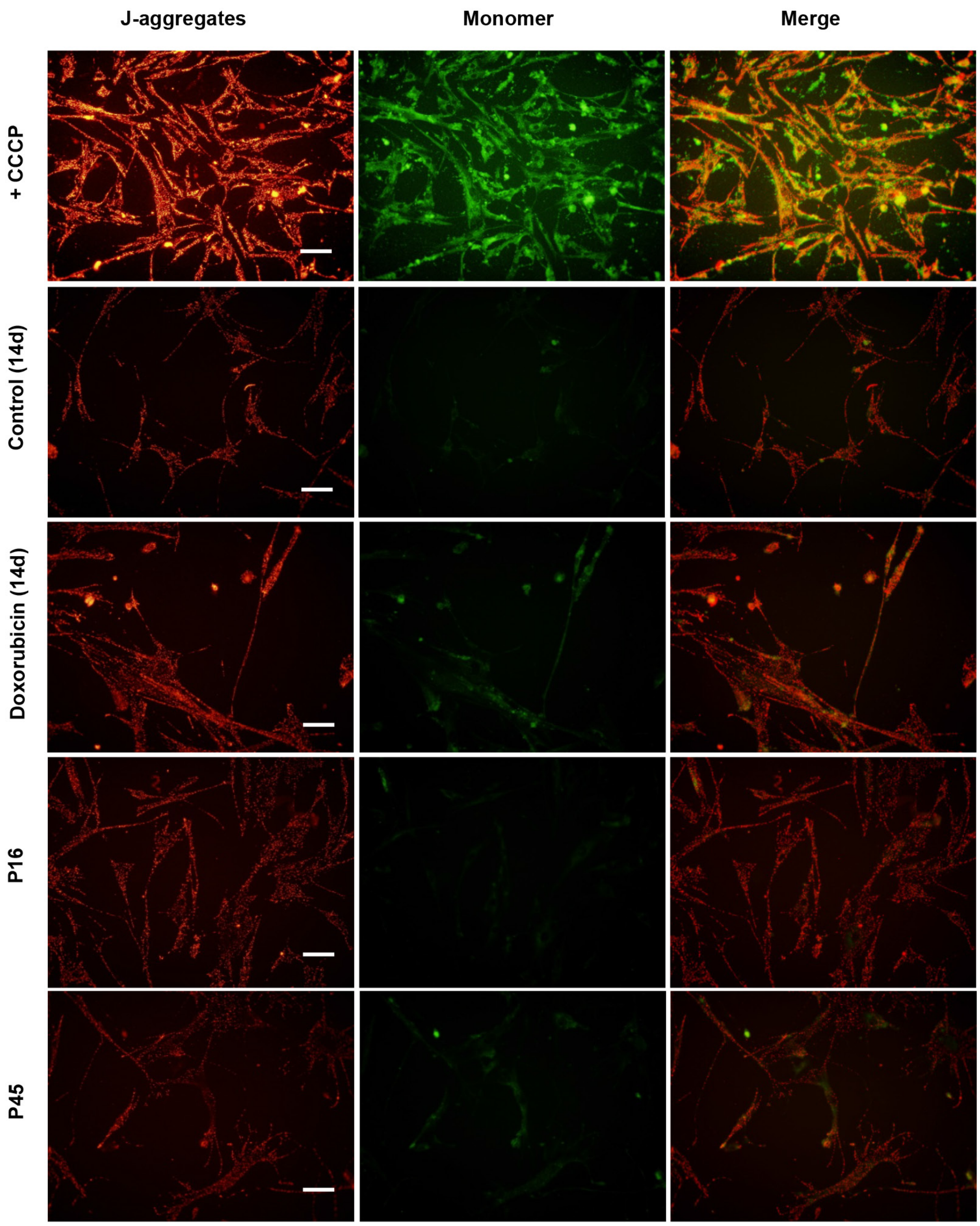

Supplementary Figure S3: MMP evaluation of senescence in TIS and RS cells before late stage. Representative fluorescence pattern after staining with JC-1 in TIS cells on day 14 and RS HFFs at P16 and P45. CCCP, an apoptosis inducer used as a positive control. Left, red fluorescence emission under green excitation corresponding to J-aggregates. Middle, green fluorescence under blue excitation which corresponding to J-monomers. Right, JC-1 images were merged by Image-J. Scale bar, 50 μm, n = 3.
